# Preparedness for use of the rapid result HIV self‐test by gay men and other men who have sex with men (MSM): a mixed methods exploratory study among MSM and those involved in HIV prevention and care

**DOI:** 10.1111/hiv.12420

**Published:** 2016-08-05

**Authors:** P Flowers, J Riddell, C Park, B Ahmed, I Young, J Frankis, M Davis, M Gilbert, C Estcourt, L Wallace, LM McDaid

**Affiliations:** ^1^ School of Health and Life SciencesGlasgow Caledonian UniversityGlasgowUK; ^2^ MRC/CSO Social and Public Health Sciences UnitUniversity of GlasgowGlasgowUK; ^3^ School of Social SciencesMonash UniversityMelbourneVic.Australia; ^4^ Applied Epidemiology UnitOntario HIV Treatment NetworkTorontoONCanada; ^5^ Barts and the London School of Medicine and DentistryBarts Sexual Health CentreBlizard InstituteLondonUK; ^6^ Health Protection ScotlandGlasgowUK

**Keywords:** HIV prevention, HIV testing, men who have sex with men, sexual health, sexual risk behaviour

## Abstract

**Objectives:**

The aim of the study was to explore preparedness for the HIV self‐test among men who have sex with men (MSM) and those involved in HIV prevention and care.

**Methods:**

A mixed methods exploratory research design was employed, detailing awareness and willingness to use the self‐test and the perceived barriers and facilitators to implementation. Quantitative and qualitative data collection and analysis were completed in parallel. Descriptive and inferential analysis of cross‐sectional bar‐based survey data collected from MSM through a self‐completed questionnaire and oral fluid specimen collection (*n* = 999) was combined with qualitative, thematic, analysis of data collected through 12 expert focus groups (*n* = 55) consisting of gay men, National Health Service (NHS) staff, community organizations, entrepreneurs and activists. Findings were subsequently combined and assessed for synergies.

**Results:**

Among MSM, self‐test awareness was moderate (55%). Greater awareness was associated with increased educational attainment [adjusted odds ratio 1.51; 95% confidence interval (CI) 1.00–2.30; *P* = 0.05] and previous history of sexually transmitted infection (STI) testing (adjusted odds ratio 1.63; 95% CI 1.11–2.39; *P* = 0.01). Willingness to use the test was high (89%) and associated with meeting sexual partners online (unadjusted odds ratio 1.96; 95% CI 1.31–2.94; *P* < 0.001). Experts highlighted the overall acceptability of self‐testing; it was understood as convenient, discreet, accessible, and with a low burden to services. However, some ambivalence towards self‐testing was reported; it could reduce opportunities to engage with wider services, wider health issues and the determinants of risk.

**Conclusions:**

Self‐testing represents an opportunity to reduce barriers to HIV testing and enhance prevention and access to care. Levels of awareness are moderate but willingness to use is high. Self‐testing may amplify health inequalities.

## Introduction

Gay and other men who have sex with men (MSM) are the group at highest risk of acquiring HIV infection within the UK. In 2013, they represented 54% of all new diagnoses. HIV testing rates amongst MSM overall have increased 1, 2 and increasing the frequency of testing to 3‐monthly for men at higher risk of HIV infection is recommended in UK national guidelines 3, 4. Scottish data for 2005–2009 showed a relatively stable incidence rate among MSM of around 15.3/1000 person‐years 5. UK data show there were 3250 new HIV diagnoses in MSM in 2013 (1) and an estimated one in five HIV positive MSM remain undiagnosed 6 with approximately 1000 late diagnoses each year 1. Delayed diagnosis is associated with poorer health outcomes and treatment response, increased mortality and health care costs, and increased levels of onward transmission 7, 8. Given that men living with HIV who are taking effective antiretroviral therapy are highly unlikely to transmit HIV 9, it is clear that undiagnosed infection, particularly primary infection (when individuals are most infectious), is responsible for most new infections 10. Mathematical modelling suggests that increased testing, linkage to care and early treatment could significantly reduce the HIV incidence in MSM 11. Furthermore, currently within the UK we know that most undiagnosed infections have occurred recently 1, and the proportion of new diagnoses associated with recent transmission has increased in some parts of the UK between 2011 to 2013 from 23% to 30% 6. In this way, getting men at high risk to test, to test regularly, and, if they test positive, to remain in care with controlled HIV is central to HIV prevention. Yet, recommendations regarding the frequency of testing are not being followed 12 and significant barriers to HIV testing endure 2. However, rapid result HIV self‐tests (or “home tests”) may offer new ways of reducing barriers to testing.

In the UK, rapid result HIV self‐test kits became legally available in April 2014, and subsequently (April 2015) commercial products became available. While self‐testing has been available in the USA for some time, it is not yet available in other national settings such as Canada, Australia or New Zealand. The international literature from countries where self‐testing has been available shows that the key facilitators to implementing, and scaling up, programmes of HIV self‐testing interventions are that they are convenient 13, 14; are quick and easy to use 15, 16; offer privacy and discretion 15, 17; are accurate and trustworthy 18; have the ability to increase knowledge of one's HIV status in resource‐limited settings 17; potentially encourage communication about HIV among potential partners 19; are acceptable to high‐risk groups 20, 21; potentially encourage more frequent testing among men with high‐risk behaviours 19; and offer immediate results 15, 22. The studies also show that key barriers to effective implementation are the lost opportunities to test for other sexually transmitted infections (STIs) 15, 23; cost issues 18, 20, 24; and perceptions of the lack of professional support available 15, 18, 24.

Here, we present findings from the first UK study of self‐testing and explore three key research questions vital for future policy and practice development in the UK with potential transferability to other international settings where HIV self‐tests may be implemented.

1. Which factors are associated with levels of awareness of the HIV self‐test among MSM?
2. Which factors are associated with willingness to use the HIV self‐test among MSM?
3. What are the key barriers and facilitators to the effective use of the rapid result HIV self‐test among the MSM population?

## Methods

Twin studies were combined and respective findings integrated to provide synergistic interpretations regarding preparedness for self‐testing across both MSM and those involved in providing HIV prevention and care services.

### Quantitative study

The University of Glasgow's triennial Gay Men's Sexual Health Survey was implemented in Glasgow, Edinburgh and Dundee in 17 venues (including two saunas) within the commercial gay scene in May 2014. Ethical approval was granted by the College of Social Sciences Ethics Subcommittee at the University of Glasgow (ref: 400130179). Data collection was similar to that in previous surveys 25, 26. Men completed an anonymous, self‐completed questionnaire and provided an oral fluid specimen (using OraSure^®^ Oral Specimen Collection Devices; OraSure Technologies, Inc, Bethlehem, PA, USA). Oral fluid specimens were analysed at the West of Scotland Specialist Virology Centre. These were tested for anti‐HIV using the Vironostika HIV Uni‐Form II Ag/Ab enzyme immunoassay (Organon Teknika, Boxtel, Netherlands). Positive samples were re‐tested and, if repeatedly reactive, were confirmed using western blot. Overall, 1340 men completed the questionnaire [45% response rate (RR)], with 1151 also providing oral fluid samples (38.6% RR). Men were excluded from the analysis if they: tested positive for HIV via the oral fluid sample (*n* = 61); did not provide a specimen (*n* = 189); had missing data on all self‐testing questions (*n* = 58); or did not self‐identify as being gay or bisexual (*n* = 33). This resulted in an overall sample size of *n* = 999.

#### The key measures included were as follows

##### Demographic and behavioural characteristics

Variables included were age, educational level and frequency of visits to the “gay scene” (i.e., bars, clubs and saunas).

##### Sexual behaviour

Respondents were asked “With how many men have you had anal sex WITHOUT a condom in the last 12 months?” and those who reported at least one unprotected anal intercourse (UAI) partner were asked: “How often was this with a casual partner?”; “How often did you know these partners' HIV status?” and “Were any of your partners HIV positive?” A single measure of higher risk sexual behaviour was derived from the above to include men who reported UAI with at least two casual, and/or unknown/discordant partners in the previous 12 months (compared with men reporting fewer than two regular, or known/concordant partners only).

##### Self‐testing awareness

Men were introduced to questions regarding the self‐test with the following text: “HIV self‐testing kits were licensed in the UK in April. This will enable men to do a test themselves and get the result immediately. This is different from self‐sampling, when you do the test at home but send the sample to a laboratory for testing.” They were then asked about awareness of self‐testing kits by answering the question “Have you heard of self‐testing kits?” with the options of yes, no or don't know. This was then recoded to “yes” (original “yes” response) and “no”/”don't know” (combined “no”/”don't know” responses).

##### Willingness to use self‐testing

Men were also asked about the likelihood of using self‐testing kits in various settings: if it was freely available on the NHS; in a community clinic or supervised location; on their own; with a partner; and if they had to pay for it. Finally, men were asked to indicate if they would be willing to use self‐testing kits under the following conditions: after a condom burst or after an episode of unprotected sex; instead of going to a clinic; before having sex with a new partner; and instead of a self‐sampling kit.

### Statistical analysis

Data were entered and analysed using spss version 21(Armonk, NY: IBM Corp.). Chi squared tests were used for bivariate comparisons and binary and multivariate logistic regression models were used to estimate odds ratios (ORs) and 95% confidence intervals (CIs) to explore factors associated with awareness of and willingness to use self‐testing kits. The final model contained all variables significant at the bivariate level (*P* < 0.05) in order to assess which remained statistically significant.

### Qualitative study

Twelve focus groups (FGs) were conducted with 55 multi‐professional, patient and provider “expert” participants between October 2014 and February 2015 in a range of settings (e.g. NHS offices, voluntary organizations and university settings). Group members were all involved in using, offering, or implementing self‐testing, or providing associated pathways into HIV care, and/or prevention. Recruitment used (1) the project funders, who assisted with local NHS recruitment within each respective health board, and (2) the research teams' existing connections with a range of organizations. Sampling balanced recruitment across urban and rural NHS board areas, and included heterogeneous groups of gay men (three FGs), a range of NHS staff (six FGs), and a range of staff from community organizations, activists and people working for businesses with vested interests in MSM (i.e. sex shop and sex sauna staff) (three FGs). An interview topic guide facilitated discussion regarding the barriers and facilitators to the implementation of self‐testing within the MSM population. Focus groups were conducted by a number of team members, primarily by CP but assisted by PF, JF, IY. Thus, facilitators were all white and included mixtures of straight, lesbian and gay researchers.

Data were transcribed and analysed thematically using nvivo 10 qualitative data analysis software (QSR International Pty Ltd. Version 10, 2012) by three of the research team (PF, BA and CP). The analytic focus was primarily descriptive, identifying areas of commonality in experts' beliefs. Ethical approval was given by Glasgow Caledonian University and NHS R and D approval for NHS Project ID: 164239; R&D2014AA089.

### Integration of findings

Following parallel and independent quantitative and qualitative data analysis, the key findings from each study were positioned within a single matrix, with a focus upon integration and synthesis. Given the differences in underlying epistemologies of each research approach (quantitative and qualitative), valuable knowledge was generated both within each study and across respective studies. As such, the matrix was interpreted by the first and last authors in relation to the complementarity and unique contribution of respective findings as patterned across various inter‐related and overlapping descriptions of context. A consensus was reached via iterative analysis and discussion. For ease of reading, the results of data integration are presented within the Discussion section of this paper.

## Results

### Quantitative study

#### Sample characteristics

The average age of participants was 34 years (range 18–82 years; standard deviation (SD) 10.96 years) with the majority identifying as gay (92.5%). Most reported post‐secondary school education (86.7%), with 34.9% reporting further/vocational‐level education, and 51.8% reporting degree/postgraduate‐level education. Almost all reported sexual contact with a man in the previous 12 months (94.0%) and 53.7% reported higher risk sexual behaviours (UAI with at least two, casual, and/or unknown/discordant partners in the previous 12 months). Only 15.4% had never had an HIV test, while 39.8% had tested in the previous 6 months and 10.1% reported having a sexually transmitted infection (STI) in the previous 12 months (Table S1, available as an online resource).

#### Factors associated with awareness of HIV self‐testing kits

Binary logistic regression compared men who had heard of self‐testing kits (*n* = 599; 60.0%) and those who had not or did not know if they had (*n* = 400; 40.0%) (Table 1). The odds of having heard of HIV self‐testing kits were significantly higher for men who identified as gay, were from Glasgow as opposed to elsewhere, reported post‐secondary school education, did not report higher risk sexual behaviours, had tested for HIV in the previous 6 months, and had ever had an STI test. When these factors were included in a multivariate logistic regression model, only having post‐secondary school education and ever having had an STI test remained significant.

**Table 1:** Demographics of those who had heard of self‐testing kits versus those who had not/did not know if they had, with unadjusted and multivariate logistic regression (*n* = 999)

	Have you heard of HIV self‐testing kits?				Unadjusted odds ratio			Adjusted odds ratio		
	Yes (*n* = 599)		No/don't know (*n* = 400)							
	*n*	%	*n*	%	OR	95% CI	*P*‐value	OR	95% CI	*P*‐value
Sexual orientation										
Gay	562	60.8	362	39.2	1		1			
Bisexual	37	49.3	38	50.7	0.63	0.39–1.01	0.05	0.78	0.46–1.34	0.37
Age										
<25 years	144	55.6	115	44.4	1					
26–35 years	221	62.6	132	37.4	1.34	0.96–1.85	0.08			
36–45 years	135	63.4	78	36.6	1.38	0.95–2.00	0.09			
≥ 46 years	94	55.6	75	44.4	1.00	0.68–1.48	1.00			
Area of residence										
Glasgow	236	64.1	132	35.9	1			1		
Edinburgh	178	58.6	126	41.4	0.79	0.58–1.08	0.14	0.82	0.58–1.17	0.28
Elsewhere	165	56.5	127	43.5	0.73	0.53–1.00	0.05	0.71	0.50–1.01	0.06
Post secondary school education										
No	58	51.3	55	48.7	1			1		
Yes	457	62.2	278	37.8	1.56	1.05–2.32	0.03	1.51	1.00–2.30	0.05
Employment status										
Not employed	95	56.9	72	43.1	1					
Employed	502	60.8	324	39.2	1.17	0.84–1.64	0.35			
Commercial gay scene use										
Low use	362	60.1	240	39.9	1					
High use	232	59.5	158	40.5	0.97	0.75–1.26	0.84			
Do you ever go online/use an app to meet sexual partners?										
No	274	57.7	201	42.3	1					
Yes	324	62.4	195	37.6	1.22	0.95–1.57	0.13			
Higher risk sexual behaviour in previous 12 months^a^										
No	298	64.4	165	35.6	1			1		
Yes	301	56.2	235	43.8	0.71	0.55–0.92	0.01	0.81	0.60–1.08	0.15
Number of HIV tests in previous 2 years										
< 4	417	60.5	272	39.5	1					
≥ 4	132	66.7	66	33.3	1.30	0.94–1.82	0.12			
More recent HIV test										
Not in last 6 months	337	56.4	260	43.6	1			1		
In last 6 months	257	65.1	138	34.9	1.44	1.10–1.87	0.01	1.22	0.89–1.67	0.21
STI in previous 12 months										
No	529	59.5	360	40.5	1					
Yes	66	66.0	34	34.0	1.32	0.86–2.04	0.21			
Ever had STI test										
No	86	49.1	89	50.9	1			1		
Yes	507	63.1	296	36.9	1.77	1.28–2.46	<0.001	1.63	1.11–2.39	0.01

CI, confidence interval; OR, odds ratio; STI, sexually transmitted infection.

a Unprotected anal intercourse with at least two, casual, and/or unknown/discordant partners in the previous 12 months.

#### Factors associated with willingness to use HIV self‐testing kits

**Figure 1:**
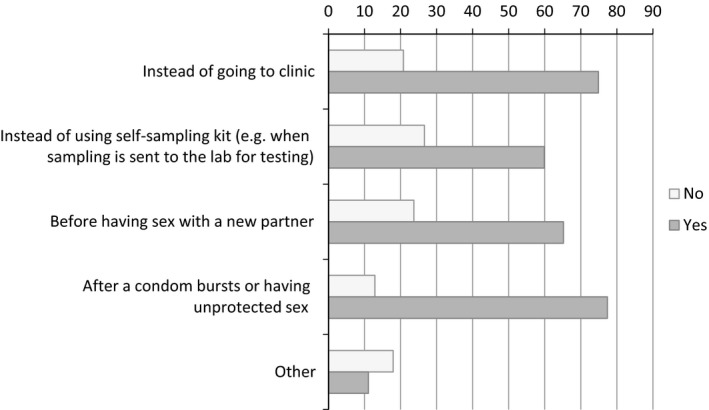
Percentages of those who would self‐test by circumstance in which they would self‐test (*n* = 887).

The majority of men (*n* = 887; 88.8%) reported that they would be willing to use HIV self‐testing kits in at least one circumstance. Of these men, 77.3% (*n* = 686) reported that they would use a kit after a condom burst or after an episode of unprotected sex, 74.9% (*n* = 664) would use a kit instead of going to a clinic, 65.2% (*n* = 578) would use a kit before having sex with a new partner, and 59.9% (*n* = 531) would use one instead of a self‐sampling kit (Fig. 1).

**Figure 2:**
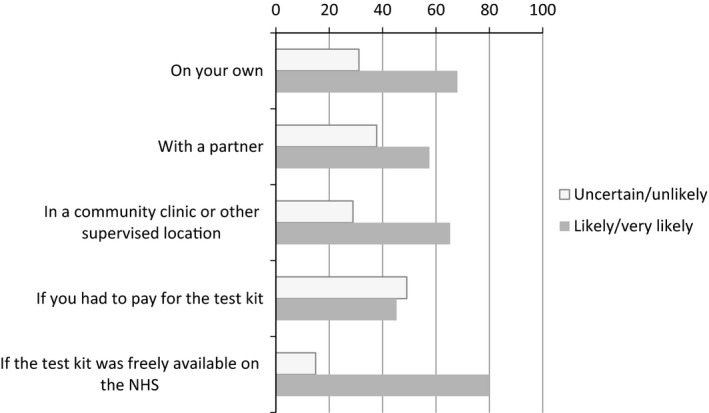
Percentages of those who would self‐test by likelihood of setting in which they would self‐test (*n* = 887).

Men were also asked about the likelihood of using self‐testing kits in various settings. Of those who were willing to self‐test in least once circumstance (*n* = 887), 79.9% (*n* = 709) reported that they were very likely/likely to use self‐testing kits if they were freely available on the NHS, 68.0% (*n* = 603) were very likely/likely to use the kit on their own, 65.3% (*n* = 579) were very likely/likely to use the kit in a community clinic or supervised location, 57.5% (*n* = 510) were very likely/likely to use the kit with a partner, and 45.2% (*n* = 401) were very likely/likely to use the kit if they had to pay for it (Fig. 2).

The characteristics of men who reported that they would be willing to use self‐testing kits are shown in Table 2. Men who reported that they ever went online/used an app to meet sexual partners were significantly more likely to report that they were willing to use self‐testing kits. There were no other significant demographic, testing, or sexual risk behaviour differences between men who were and were not willing to use self‐testing kits.

**Table 2:** Demographics and unadjusted odds ratios of those who would use self‐testing kits versus those who would not (*n* = 999)

	Would use self‐testing kits				Unadjusted Odds Ratio		
	Yes		No				
	*n*	(%)	*n*	%	OR	95% CI	*P*‐Value
Sexual Orientation							
Gay	819	88.6	105	11.4	1		
Bisexual	68	90.7	7	9.3	1.25	0.56–2.78	0.59
Age							
< 25	236	91.1	23	8.9	1		
26–35	311	88.1	42	11.9	0.72	0.42–1.23	0.23
36–45	187	87.8	26	12.2	0.70	0.39–1.27	0.24
46+	150	88.8	19	11.2	0.77	0.41–1.46	0.42
Area of residence							
Glasgow	326	88.6	42	11.4	1		
Edinburgh	269	88.5	35	11.5	0.99	0.61–1.60	0.97
Elsewhere	263	90.1	29	9.9	1.17	0.71–1.93	0.54
Post secondary school education							
No	99	87.6	14	12.4	1		
Yes	652	88.7	83	11.3	1.11	0.61–2.03	0.73
Employment status							
Not employed	152	91.0	15	9.0	1		
Employed	730	88.4	96	11.6	0.75	0.42–1.33	0.32
Commercial gay scene use							
Low Use	533	88.5	69	11.5	1		
High Use	347	89.0	43	11.0	1.04	0.70–1.56	0.83
Ever go online/use an app to meet sexual partners							
No	405	85.3	70	14.7	1		
Yes	477	91.9	42	8.1	1.96	1.31–2.94	<0.001
Higher risk sexual behaviour in previous 12 months*							
No	405	87.5	58	12.5	1		
Yes	482	89.9	54	10.1	1.28	0.86–1.89	0.22
Number of HIV tests in previous 2 years							
<4	610	88.5	79	11.5	1		
4+	174	87.9	24	12.1	0.94	0.58–1.53	0.80
More recent HIV test							
Not in last 6 months	536	89.8	61	10.2	1		
In last 6 months	345	87.3	50	12.7	0.79	0.53–1.17	0.23
STI in previous 12 months							
No	786	88.4	103	11.6	1		
Yes	93	93.0	7	7.0	1.74	0.79–3.86	0.17
Ever had STI test							
No	160	91.4	15	8.6	1		
Yes	711	88.5	92	11.5	0.72	0.41–1.28	0.27

CI, confidence interval; OR, odds ratio; STI, sexually transmitted infection.

*Unprotected anal intercourse with at least two, casual, and/ or unknown/discordant partners in the previous 12 months.

### Exploratory qualitative study

Table 3 illustrates the major themes that the participants raised concerning the key facilitators and barriers to the uptake and use of the self‐test.

**Table 3:** Overview of perceived barriers and facilitators to self‐testing among men who have sex with men (MSM)

Facilitators to the uptake and use of the self‐test	Barriers to the uptake and use of the instant result HIV self‐test
Consensus regarding Convenience/Speed of testing and accessing test results	Provider perceptions of the lost opportunities for engagement with range of services and staff
Consensus regarding perceptions of high discretion and privacy	Consensus regarding concerns relating to deracinating HIV from wider and holistic health
Provider perceptions of the need to rationalise clinical time and resources	Consensus regarding perceived negative consequences of receiving reactive test results (suicide, distress, isolation)
Consensus regarding the test's ability to reach new and potentially vulnerable populations	MSM perceptions of poor trust and low perceived accuracy of the kit
MSM perceptions that self‐testing avoided the stigma of utilising GUM services	Consensus regarding high levels of health literacy and skills required to use the kit correctly

GUM, genitourinary medicine.

#### Facilitators to uptake and use of rapid result HIV self‐test

The convenience, immediacy, discretion and privacy of testing within one's own home were important factors in facilitating the likely implementation of the self‐test and understanding how the self‐test could reduce barriers to testing. Autonomy regarding decisions on when to test and dramatically reduced waiting times for results figured strongly within the discussions as other key facilitators. Self‐testing was welcomed in terms of cost‐efficiency and the rationalization of limited NHS resources; for example, “how expensive is it to post a kit as opposed to half an hour of a consultant's time?” (NHS staff FG). Self‐testing was also described as potentially reaching new populations by reducing perceived barriers to testing, such as the stigma associated with use of traditional genitourinary medicine (GUM) services:

P7 *…*there are people who don't engage with sexual health services, so anything that maybe makes them test would surely be worthwhile, for these people who will never engage with us.

P2 Yes, it's still got a terrible stigma, it's still the clap clinic, people come in they were like, “Oh it's awful in the waiting room” and “I hate being here”, “I never thought I would have to come to a place like this”, and people always say stuff like that.

(NHS staff FG)

Discussions also detailed how the self‐test facilitated testing and would reduce barriers to testing by accommodating the hectic reality of many people's lives. It could be easily used by men who had busy lives, who lived rurally, and who would struggle to use traditional clinics for a range of reasons: “I think it's good, because some people, for example, they're married and they have children, for example, some men. But they still engage, like, in gay sex. Obviously, they're not going to their doctor to get a test, a blood test” (Gay men's FG). In this way, the self‐test could enable high‐risk men to test more frequently and could enable more vulnerable men to engage in testing for the first time. For example, “So what better way of actually taking away any stigma about it than to have something as regular where you might actually go to pick up paracetamol?” (Non‐NHS stakeholder FG).

#### Barriers to uptake and use of rapid result HIV self‐test

Key barriers to the uptake and use of self‐testing focussed upon lost opportunities to engage with a wide range of services, staff and holistic understandings of health and the actual determinants, as opposed to the consequences, of risk behaviour, as the following extract shows: “very rarely is it just about… When someone goes to, even a sexual health clinical, very rarely is it just about sexual health. There are other support needs” (Non‐NHS stakeholder FG). Primarily among NHS staff, there was some resistance to the idea that the decoupling of testing from traditional services was beneficial or warranted. Participants outlined the lost opportunities that self‐testing technologies herald; there is no guaranteed continuity of care, no easy access to the full range of clinical expertise (e.g. in mental health, wellbeing, relationship concerns, drugs, and alcohol), no partner notification, and no opportunity for additional risk reduction and intervention. Similarly, major concerns were articulated about the test users discovering their reactive results when alone.

F1 Uh‐huh. What are your concerns, P2?

P2 Just the one o'clock in the morning stuff.

P1 I know, and go and jump off a bridge or something.

P2 Yeah, and I suppose that is…

P1 That is the worry.

P2 …I suppose that is the difference between home testing and home sampling, is, okay, you've got the advantage that you've got it within 20 minutes, half an hour, whatever it might be, which is great. The disadvantage is just that lack of connection with other people, and that support.

(NHS staff rural areas)

The discussions also outlined barriers to the effective use of self‐testing in relation to health inequalities and health literacy. Key concerns focussed upon the skills, abilities and knowledge levels among those who were testing and their accurate interpretation of test results:

P1 The test is going back to the timing from infection date to it being shown, showing up in your blood. So, I think the errors that can be involved in self‐testing are with yourself, you know, wait until you, what do you call it, your viral load becomes testable. There's no like pregnancy wait for two weeks […..].

P4 I think this probably then also depends on how educated you are about it in the first place, rather than, okay, you know everything about it, so we'll take a test and that's your result. It depends on how educated you are about when your potential exposure was

(Gay men rural area)

## Discussion

This work has explored preparedness for self‐testing and describes the overall acceptability of the self‐test in the UK for the first time. The triangulation and integration of key constructs from the two constitutive studies reported here are shown in Table 4. This suggests the overall acceptability of the self‐test and highlights its potential to increase HIV testing among some, but not all, MSM. It also suggests that the optimization and subsequent effective implementation of self‐testing will change HIV prevention policy and practice. In turn, this will have an impact upon how HIV care and surveillance should also be considered.

**Table 4:** Integration of the main findings across the constitutive studies

Key contexts	Quantitative study	Qualitative study	Interpretation and synthesis
Technological level – the acceptability of the self‐test	Although awareness was low, willingness to use self‐tests was high	Self‐tests were considered to reduce barriers to testing and have the ability to reach new and potentially vulnerable populations	Self‐tests are a tool with the potential to increase testing
Individual level	Awareness of self‐testing was associated with post‐secondary school education and ever having had an STI test. The self‐test offers new opportunities for self‐management of HIV risks, with 77.3% reporting that they would use a kit after a condom burst or after an episode of unprotected sex and 65.2% reporting that they would use a kit before having sex with a new partner	Using the kit correctly was regarded to require high levels of health literacy and skills, and perceptions of poor trust and low perceived accuracy of the kit were expressed by MSM. While self‐tests were perceived to offer high discretion and privacy, there are potentially negative consequences of receiving reactive test results alone (suicide, distress and isolation)	Health and HIV literacy is important in terms of using and understanding the results of the kit. There is also potential vulnerability of men receiving reactive results on their own and risk of men being misinformed by taking the self‐test at the wrong time (i.e. immediately after a risk event)
Service/community evel	Self‐tests offer an alternative to clinic testing, with 74.9% reporting that they would use a kit instead of going to a clinic. While 68.0% were likely to use the kit on their own, 57.5% reported that they were likely to use the kit with a partner and 65.3% were likely to use the kit in a community clinic or supervised location	There was consensus among stakeholders regarding the convenience and speed of testing and accessing results and, for MSM, avoiding the perceived stigma of using GUM services. However, providers highlighted lost opportunities for engagement with a range of services and staff	The self‐test could lead to a lack of engagement with traditional NHS services, but could be provided in alternative community settings to relieve pressure on the NHS. Testing between partners could facilitate discussions on HIV status, but this could leave men at risk of violence and abuse
Social level	Most men (79.9%) reported that they were likely to use self‐tests if they were freely available on the NHS, while only 45.2% were willing to pay for the tests. Willingness to use the self‐test was only associated with the use of the internet and phone apps to meet sexual partners	Additional social and geographical factors, such as relative isolation, may also influence who might be more likely to use the test and digital media. Although self‐tests could relieve pressures on clinical time and resources, deracinating HIV from wider and holistic health is contrary to the existing policy landscape	The social and economic context in which self‐tests are provided, and existing inequalities among MSM, are likely to shape uptake, but a move towards self‐testing could have unintended consequences for broader sexual health and wellbeing by amplifying health inequalities

At the individual level, health and HIV literacy were important, with awareness of self‐testing associated with level of educational attainment. Concerns were also expressed about the accuracy of interpreting test results in relation to the window period and to specific risk events. Preferences to use the self‐test rather than visiting a clinic or using self‐sampling, combined with perceptions of convenience and ease of use and potential reductions in stigma, suggest that it is highly likely that the self‐test will reduce some barriers to testing. However, use of the self‐test may therefore lead to a lack of engagement with traditional NHS services. While this may rationalize limited NHS resources, it was also concerning from both the patient and provider perspectives, particularly in relation to follow‐up of reactive results and the accuracy of epidemiological surveillance, but also in relation to lost opportunities for prevention such as interventions that address the determinants of on‐going risk behaviour, or partner notification.

The synthesis also indicates the likely importance of social and economic factors in shaping the kinds of men who may use the self‐test. It illuminates the complexity of these issues; the quantitative findings representing bar‐based urban populations of MSM suggest the potential importance of digital and social media use in the likelihood of using the self‐test, with concomitant repercussions extending to service redesign for accessing future regular testing, the provision of prevention interventions, accessing confirmatory testing, and on‐going care for those who test positive. These findings could reflect the role of digital literacy, or a propensity to engage with innovation or online consumerism; yet, tellingly, patterns in likelihood of use also relate to whether the test would be provided free by the NHS. The qualitative findings, which reflect perspectives from both urban and rural areas, highlighted how additional social and geographical factors, such as relative isolation, may also influence who might be more likely to use both the test and indeed the digital media. Although self‐tests could relieve pressures on clinical time and resources, deracinating HIV from wider and holistic health is contrary to the existing policy landscape and could have unintended consequences for broader sexual health and wellbeing.

### Strengths and weaknesses

The strengths of this study were its originality and its effective use of exploratory mixed methods. These maximized the strengths of the qualitative analysis which both captured the complexity of the issue and inductively identified areas of concern (e.g. the amplification of health inequities). The study also consolidated the benefits of quantitative research approaches, illuminating population means and other nomothetic insights (e.g. apparent preferences for self‐testing over self‐sampling). Its weaknesses relate to its exploratory cross‐sectional design and its geographical reach (Scotland only), its sole focus upon the MSM population rather than other populations such as black Africans in the UK, its use of a sample of MSM who mostly identified as gay and were highly educated, and the temporal collection of data prior to self‐testing products becoming commercially available. Recent surveys of MSM recruited via other approaches also suggest that our sampling strategies may well oversample those already engaged with testing behaviours *per se*; for example, only 52% of MSM in the general population report having an HIV test within the past 5 years and comparison of population‐based probability surveys and venue‐based convenience surveys has shown increased recent testing in the latter 27, 28. Self‐testing may well be more relevant for MSM populations not currently using traditional testing services or enrolling in venue‐based convenience samples and our work here may underestimate the potential value of self‐testing. The findings have international relevance for countries with similar epidemics among MSM where the self‐test has yet to be made available (e.g. Canada) or where it is currently becoming available (e.g. New Zealand and Australia).

### Importance and implications

Self‐testing represents a relatively new development within the HIV prevention and care tool kit. Our integrated exploratory findings anticipate that it has great potential to shape HIV prevention and care. Yet, our findings also suggest that its potential will not be realized if it is not considered in relation to the heterogeneity of MSM and the diversity of their needs and preferences. Self‐testing has the capacity to be a transformative technology, potentially, a core part of an integrated online HIV prevention and care system for men who choose this approach and have the requisite digital and health literacy [this could combine access to pre‐exposure prophylaxis (PrEP) and other behavioural prevention initiatives]. However, our integrated analysis also suggests the particular vulnerability of some MSM populations and the potential for further isolation if shifts to digital technologies and self‐managed diagnostic testing reduce provision for other testing opportunities.

### Further research

Further research must examine self‐testing at the individual, organisational and social levels. For example, at the individual level, who will use it? Why do they choose it, rather than, for example, self‐sampling, and indeed which type of self‐test do they prefer (blood or oral)? How will they use it? When, in which circumstances, and with what consequences (e.g. delayed confirmation, “loss to follow‐up” or suicide) will they use it? At the organizational level, questions of the effectiveness and cost‐effectiveness of self‐testing become important; how can it be optimized to reduce barriers to testing? Can it reduce undiagnosed infection and late diagnosis? What, if any, are the health economic benefits of self‐testing and how can they be enhanced (e.g. by targeted self‐testing)? How should the NHS and other services be redesigned to accommodate the way in which self‐testing is decoupled from existing services? To what extent can online prevention and care services complement the self‐management of these diagnostic technologies, and would such online services improve patient experience and contribute to health improvements? With regard to the social consequences of self‐testing, research is needed that examines how self‐testing may amplify health inequities, enabling MSM with good health and digital literacy skills to improve their health while investment in more traditional services and their service users (e.g. with poorer health and digital literacy) reduces.

## Author contributions

PF and LM devised the study, and PF and JR wrote the first draft of the manuscript. PF led the qualitative analysis with assistance from CP and BA; JR undertook quantitative data analysis and contributed to drafts of the manuscript. PF and LM integrated the data analysis. All authors contributed to interpretation of the data, contributed revisions, and approved the final version of the manuscript.

## Supporting information


**Table S1.** Sample characteristics of men (*n* = 999).

## References

[1] Yin Z , Brown A , Hughes G , Nardone A , Gill O , Delpech V . HIV in the United Kingdom 2014 report: data to end 2013. London, Public Health England, 2014.

[2] Flowers P , Knussen C , Li J , McDaid L . Has testing been normalized? An analysis of changes in barriers to HIV testing among men who have sex with men between 2000 and 2010 in Scotland, UK. HIV Med 2013; 14: 92–98. 22934820 10.1111/j.1468-1293.2012.01041.x PMC3561706

[3] BASHH . Recommendations for Testing for Sexually Transmitted Infections in Men‐who have Sex‐with‐Men. London, BASHH, 2014.

[4] NSWSPU . Australian sexually transmitted infection & HIV testing guidelines 2014 for asymptomatic men who have sex with men. Sydney; 2014.

[5] McDonald SA , Hutchinson SJ , Wallace LA *et al* Trends in the incidence of HIV in Scotland, 1988–2009. Sex Transm Infect 2012; 88: 194–199. 22158935 10.1136/sextrans-2011-050132

[6] Aghaizu A , Brown AE , Nardone A *et al* HIV in the United Kingdom 2013 Report: Data to End 2012. London: Public Health England, 2013.

[7] May M , Gompels M , Delpech V *et al* Impact of late diagnosis and treatment on life expectancy in people with HIV‐1: UK Collaborative HIV Cohort (UK CHIC) Study. BMJ 2011; 343: d6016. 21990260 10.1136/bmj.d6016 PMC3191202

[8] Nakagawa F , Lodwick RK , Smith CJ *et al* Projected life expectancy of people with HIV according to timing of diagnosis. AIDS 2012; 26: 335–343. 22089374 10.1097/QAD.0b013e32834dcec9

[9] Cohen MS , McCauley M , Gamble TR . HIV treatment as prevention and HPTN 052. Curr Opin HIV AIDS 2012; 7: 99. 22227585 10.1097/COH.0b013e32834f5cf2 PMC3486734

[10] Skarbinski J , Rosenberg E , Paz‐Bailey G *et al* Human immunodeficiency virus transmission at each step of the care continuum in the United States. JAMA Intern Med 2015; 175: 588–596. 25706928 10.1001/jamainternmed.2014.8180

[11] Phillips AN , Cambiano V , Nakagawa F *et al* Increased HIV incidence in men who have sex with men despite high levels of ART‐induced viral suppression: analysis of an extensively documented epidemic. PLoS One 2013; 8: e55312. 23457467 10.1371/journal.pone.0055312 PMC3574102

[12] McDaid L , Aghaizu A , Frankis J *et al* Frequency of HIV testing among gay and bisexual men in the UK: implications for HIV prevention. HIV Med 2016; doi:10.1111/hiv.12373 10.1111/hiv.12373 PMC5026165 26991460

[13] Pant Pai N , Sharma J , Shivkumar S *et al* Supervised and unsupervised self‐testing for HIV in high‐ and low‐risk populations: a systematic review. PLoS Med 2013;10:e1001414. 23565066 10.1371/journal.pmed.1001414 PMC3614510

[14] Young SD , Daniels J , Chiu CJ *et al* Acceptability of using electronic vending machines to deliver oral rapid HIV self‐testing kits: a qualitative study. PLoS One 2014; 9: e103790. 25076208 10.1371/journal.pone.0103790 PMC4116256

[15] Bilardi J , Walker S , Read T *et al* Gay and bisexual men's views on rapid self‐testing for HIV. AIDS Behav 2013; 17: 2093–2099. 23297083 10.1007/s10461-012-0395-7

[16] Lee VJ , Tan SC , Earnest A , Seong PS , Tan HH , Leo YS . User acceptability and feasibility of self‐testing with HIV rapid tests. J Acquir Immune Defic Syndr 1999; 2007 : 449–453. 10.1097/QAI.0b013e318095a3f3 17554213

[17] Cambiano V , Mavedzenge SN , Phillips A . Modelling the potential population impact and cost‐effectiveness of self‐testing for HIV: evaluation of data requirements. AIDS Behav 2014; 18 (Suppl 4): 450–458. 10.1007/s10461-014-0824-x PMC4094791 24957978

[18] Wood BR , Ballenger C , Stekler JD . Arguments for and against HIV self‐testing. HIV/AIDS (Auckland, NZ). 2014;6:117–126. 10.2147/HIV.S49083 PMC4126574 25114592

[19] Carballo‐Dieguez A , Frasca T , Balan I , Ibitoye M , Dolezal C . Use of a rapid HIV home test prevents HIV exposure in a high risk sample of men who have sex with men. AIDS Behav 2012; 16: 1753–1760. 22893194 10.1007/s10461-012-0274-2 PMC3458207

[20] Katz DA , Golden MR , Hughes JP , Farquhar C , Stekler JD . Acceptability and ease of use of home self‐testing for HIV among men who have sex with men. 19th Conference on Retroviruses and Opportunistic Infections. Seattle, WA., 2012.

[21] Carballo‐Dieguez A , Frasca T , Dolezal C , Balan I . Will gay and bisexually active men at high risk of infection use over‐the‐counter rapid HIV tests to screen sexual partners? J Sex Res 2012; 49: 379–387. 22293029 10.1080/00224499.2011.647117 PMC3600862

[22] Bavinton B , Brown G , Hurley M *et al* Which gay men would increase their frequency of hiv testing with home self‐testing? AIDS Behav 2013; 17: 2084–2092. 23525790 10.1007/s10461-013-0450-z

[23] Cabié A , Bissuel F , Huc P , Paturel L , Abel S . Impact of rapid HIV testing on the return rate for routine test results in sexually transmitted infection testing centres. Int J STD AIDS 2011; 22: 757–758. 22174063 10.1258/ijsa.2009.009267

[24] Ng OT , Chow AL , Lee VJ *et al* Accuracy and user‐acceptability of hiv self‐testing using an oral fluid‐based HIV rapid test. PLoS One 2012; 7: e45168. 23028822 10.1371/journal.pone.0045168 PMC3444491

[25] McDaid LM , Hart GJ . Increased HIV testing and reduced undiagnosed infection among gay men in Scotland, 2005–8: support for the opt‐out testing policy? Sex Transm Infect 2011; 87: 221–224. 21325443 10.1136/sti.2010.044560 PMC3791475

[26] Wallace LA , Li J , McDaid LM . HIV Prevalence and undiagnosed infection among a community sample of gay and bisexual men in Scotland, 2005‐2011: implications for HIV testing policy and prevention. PLoS One 2014; 9: e90805. 24621479 10.1371/journal.pone.0090805 PMC3951276

[27] Sonnenberg P , Clifton S , Beddows S *et al* Prevalence, risk factors, and uptake of interventions for sexually transmitted infections in Britain: findings from the National Surveys of Sexual Attitudes and Lifestyles (Natsal). Lancet 2013; 382: 1795–1806. 24286785 10.1016/S0140-6736(13)61947-9 PMC3899025

[28] Prah P , Hickson F , Bonell C *et al* Men who have sex with men in Britain: comparison of estimates from a probability sample and community‐based surveys. Sex Transm Infect In press; doi:10.1136/sextrans‐2015‐052389 10.1136/sextrans-2015-052389 PMC5013102 26965869

