# Supplementary material for: Preparedness for use of the rapid result HIV self‐test by gay men and other men who have sex with men (MSM): a mixed methods exploratory study among MSM and those involved in HIV prevention and care
Source: HIV Med. 2016 Aug 5;18(4):245–55. doi: 10.1111/hiv.12420 (PMC5347967; doi:10.1111/hiv.12420)
Supplement: Supplementary file 1 — Table S1. Sample characteristics of men (n = 999). [file HIV-18-245-s001.docx]

**Table S1: Sample characteristics of men (n=999)**

|  | **Total** | |
| --- | --- | --- |
|  | **n** | **%** |
| **Sexual Orientation** | | |
| **Gay** | 924 | 92.5 |
| **Bisexual** | 75 | 7.5 |
| **Age** | | |
| **<25** | 259 | 26.1 |
| **26-35** | 353 | 35.5 |
| **36-45** | 213 | 21.4 |
| **46+** | 169 | 17.0 |
| **Area of residence** | | |
| **Glasgow** | 368 | 38.2 |
| **Edinburgh** | 304 | 31.5 |
| **Elsewhere** | 292 | 30.3 |
| **Post secondary school education** | | |
| **No** | 113 | 13.3 |
| **Yes** | 735 | 86.7 |
| **Employment status** | | |
| **Not employed** | 167 | 16.8 |
| **Employed** | 826 | 83.2 |
| **Commercial Gay Scene Use** | | |
| **Low Use** | 602 | 60.7 |
| **High Use** | 390 | 39.3 |
| **Do you ever go online/ use an app to meet sexual partners?** | | |
| **No** | 475 | 47.8 |
| **Yes** | 519 | 52.2 |
| **Higher risk sexual behaviour in previous 12 months^[[1]](#footnote-1)^** |  |  |
| **No** | 463 | 46.3 |
| **Yes** | 536 | 53.7 |
| **Number of HIV tests in previous 2 years** | | |
| **<4** | 689 | 77.7 |
| **4+** | 198 | 22.3 |
| **More recent HIV test** | | |
| **Not in last 6 months** | 597 | 60.2 |
| **In last 6 months** | 395 | 39.8 |
| **STI in previous 12 months** | | |
| **No** | 889 | 89.9 |
| **Yes** | 100 | 10.1 |
| **Ever had STI test** | | |
| **No** | 175 | 17.9 |
| **Yes** | 803 | 82.1 |
|  | | |

1. UAI with 2+ casual, and/ or unknown/discordant partners in the previous 12 months [↑](#footnote-ref-1)
